# Catecholamines as outcome markers in isolated traumatic brain injury: the COMA-TBI study

**DOI:** 10.1186/s13054-017-1620-6

**Published:** 2017-02-23

**Authors:** Sandro B. Rizoli, Blessing N. R. Jaja, Alex P. Di Battista, Shawn G. Rhind, Antonio Capone Neto, Leodante da Costa, Kenji Inaba, Luis Teodoro da Luz, Bartolomeu Nascimento, Adic Perez, Andrew J. Baker, Airton Leonardo de Oliveira Manoel

**Affiliations:** 1 St. Michael’s Hospital, 30 Bond Street, Toronto, ON M5B 1W8 Canada; 2 Keenan Research Centre for Biomedical Science of St. Michael’s Hospital, 30 Bond Street, Toronto, ON M5B 1W8 Canada; 3 Institute of Medical Science, University of Toronto, Toronto, ON Canada; 4 Defence Research and Development Canada (DRDC), Toronto Research Centre, Toronto, ON Canada; 5 Hospital Israelita Albert Einstein, Avenida Albert Einstein, 627/701 - Morumbi, São Paulo, SP 05652-900 Brazil; 6 Department of Surgery, Sunnybrook Health Sciences Centre, University of Toronto., 2075 Bayview Ave., Toronto, ON M4N 3M5 Canada; 7 University of Southern California, 2051 Marengo Street, IPT, C5L100, Los Angeles, CA 90033 USA; 8 Neuroscience Research Program, Keenan Research Centre of the Li Ka Shing Knowledge Institute, St. Michael’s Hospital, 30 Bond Street, Toronto, ON M5B 1W8 Canada

**Keywords:** Traumatic brain injury, Catecholamines, Epinephrine, Norepinephrine, Functional outcome

## Abstract

**Background:**

Elevated catecholamine levels might be associated with unfavorable outcome after traumatic brain injury (TBI). We investigated the association between catecholamine levels in the first 24 h post-trauma and functional outcome in patients with isolated moderate-to-severe TBI.

**Methods:**

A cohort of 174 patients who sustained isolated blunt TBI was prospectively enrolled from three Level-1 Trauma Centers. Epinephrine (Epi) and norepinephrine (NE) concentrations were measured at admission (baseline), 6, 12 and 24 h post-injury. Outcome was assessed at 6 months by the extended Glasgow Outcome Scale (GOSE) score. Fractional polynomial plots and logistic regression models (fixed and random effects) were used to study the association between catecholamine levels and outcome. Effect size was reported as the odds ratio (OR) associated with one logarithmic change in catecholamine level.

**Results:**

At 6 months, 109 patients (62.6%) had an unfavorable outcome (GOSE 5–8 vs. 1–4), including 51 deaths (29.3%). Higher admission levels of Epi were associated with a higher risk of unfavorable outcome (OR, 2.04, 95% CI: 1.31–3.18, *p* = 0.002) and mortality (OR, 2.86, 95% CI: 1.62–5.01, *p* = 0.001). Higher admission levels of NE were associated with higher risk of unfavorable outcome (OR, 1.59, 95% CI: 1.07–2.35, *p* = 0.022) but not mortality (OR, 1.45, 95% CI: 0.98–2.17, *p* = 0.07). There was no relationship between the changes in Epi levels over time and mortality or unfavorable outcome. Changes in NE levels with time were statistically associated with a higher risk of mortality, but the changes had no relation to unfavorable outcome.

**Conclusions:**

Elevated circulating catecholamines, especially Epi levels on hospital admission, are independently associated with functional outcome and mortality after isolated moderate-to-severe TBI.

**Electronic supplementary material:**

The online version of this article (doi:10.1186/s13054-017-1620-6) contains supplementary material, which is available to authorized users.

## Background

Traumatic brain injury (TBI) is the leading cause of disability and mortality among young adults worldwide, with a major socio-economic impact and costs of more than US$60 billion per year in the USA alone [1–3]. Trauma elicits a complex systemic response, characterized by profound alterations in neuroendocrine and immune function geared toward restoring homeostasis [4]. Activation of the hypothalamic-pituitary-adrenal axis and the sympathetic nervous system (SNS) leads to the secretion of glucocorticoids and catecholamines respectively, along with complex neuroimmune interactions [4]. These changes are recognized as central pathways in the pathogenesis of post-traumatic complications [4, 5]. Traumatic brain injury, in particular, leads to immediate and profound SNS activation with massive release of catecholamines [epinephrine (Epi), norepinephrine (NE)] [6]. While the adrenergic response is essential for survival – hypotension doubles mortality of patients with severe TBI [7] – it also increases oxygen demand by the heart and brain causing cardiovascular dysfunction and may lead to further brain damage. We hypothesized that in patients with moderate-to-severe TBI, elevated levels of circulating catecholamines measured on admission are associated with unfavorable 6-month functional outcome. Therefore, we conducted a prospective, observational cohort study to evaluate the association between circulating catecholamine levels and functional outcome after isolated blunt moderate-to-severe TBI.

## Methods

### Selection of participants

A prospective, observational cohort study was conducted in three Level-1 Trauma Centers, two centers in Canada and one center in USA, from November 2011 to September 2013. Inclusion criteria: (a) adult patients (age ≥ 16 years); (b) isolated blunt moderate-to-severe TBI, defined by a Glasgow Coma Scale [8] (GCS) score <13; and (c) non-head Abbreviated Injury Scores (AIS) ≤ 2. Exclusion criteria included: (a) elapsed time between injury and admission to the Emergency Department (ED) exceeding 3 hours; (b) age <16 years; (c) pregnancy; (d) absence of vital signs prior to ED admission; and (e) penetrating head injury.

**Fig. 1:**
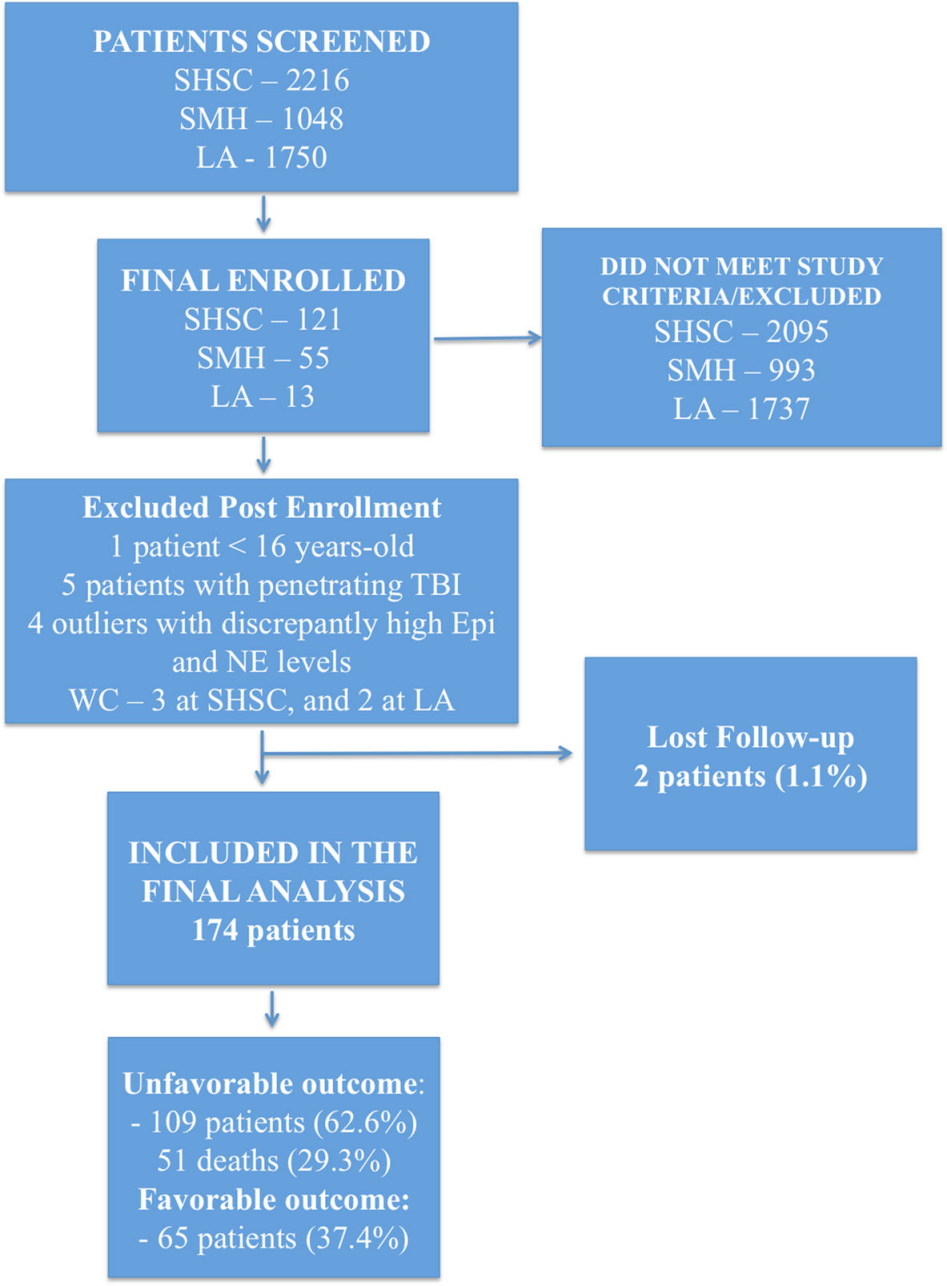
Flow diagram of the screening process. *LA* Los Angeles County General Hospital and the University of Southern California Medical Center, *SHSC* Sunnybrook Health Sciences Centre, *SMH* St. Michael’s Hospital, *WC* withdrawal of consent

Figure 1 illustrates the study enrollment process and follow-up according to the STROBE statement: guidelines for reporting observational studies [9]. From September 2011 to June 2013, 3264 patients were screened in two trauma centers in Toronto [2216 at Sunnybrook Health Sciences Centre (SHSC), 1048 at St. Michael’s Hospital (SMH)] and from January 2013 to March 2013, 1750 patients were screened at the LA County General Hospital and the University of Southern California Medical Center (LA County). Two thousand and ninety-five patients did not meet the inclusion criteria at SHSC, 978 at SMH and 1737 at LA County. The final cohort was of 189 patients with isolated moderate-to-severe TBI enrolled in the study (121 patients from SHSC, 55 patients from SMH and 13 patients from LA County). In total 15 patients were excluded from the final analysis: one patient was excluded later after enrollment, because it was noticed that the age was less than 16 years old; three patients at SHSC and four patients at LA County were removed from the cohort due to withdrawal of consent post enrollment, by the patient’s power of attorney. Additionally, five patients with penetrating TBI, and four outliers with discrepantly high catecholamine levels were also excluded. The outliers had levels that were above the upper limits of detection of the assay. Two patients were not located using previous contact information and within the provincial registries, and we could not assess their long-term outcome.

*Control group:* following informed consent, peripheral venous blood samples were also collected once from 50 healthy volunteers [age 30.3 ± 7.7 years (mean ± SD)] using a 21-gauge needle following a resting period of 30 minutes. Their catecholamine levels were used as the control/baseline catecholamine levels. Control participants were recruited locally by advertisement and excluded if they had any previous history of TBI or co-morbidities.

### Procedures and data collection

Clinical, laboratory and imaging data were collected upon hospital arrival and throughout the hospital stay. It included baseline demographics, trauma information [i.e., mechanism of injury, elapsed time from the injury to hospital, Injury Severity Score (ISS), Abbreviated Injury Scores (AIS), computed tomography (CT) Marshall Classification [10] (Additional file 1: Table S1), laboratory values, neurological and clinical status, and past medical history]. A complete list of procedures and data collection can be found in the Additional file 1.

### Sample collection and preservation

Venous blood samples for plasma catecholamine analyses were drawn into 10-mL K_2_EDTA vacutainers (Vacutainer, Becton Dickinson, Rutherford, NJ, USA) as soon as possible after admission to the trauma room (baseline) and again at 6, 12 and 24 h post admission. Specimens were immediately centrifuged at 1600 × *g* for 15 minutes (4 °C), the plasma separated into aliquots and frozen at −70 °C until analyses. The teams caring for the patients were blinded to the results of all research assays and consequently the results were not available for treatment decisions.

#### Determination of plasma catecholamines concentrations

Plasma Epi and NE concentrations (pmol/L) were determined from duplicate samples using a direct competitive enzyme immunoassay method according to the manufacturer’s instructions (Bi-CAT EIA, Alpco Diagnostics, Salem, NH, USA). Briefly, plasma Epi and NE were extracted by using a cis-diol-specific affinity gel, acylated and then derivatized enzymatically into N-acylmetanephrine and N-acylnormetanephrine, respectively. Antibody bound to the solid-phase catecholamines was detected by an anti-rabbit IgG-peroxidase conjugate using tetramethylbenzidine as a substrate. This colorometric reaction was terminated by the addition of 0.25 M H_2_SO4 and the absorbance measured at 450 nanometers (nm) and 630 nm using a multi-detection microplate reader (VICTOR 3, PerkinElmer, Waltham, MA, USA). Quantification of unknown samples was achieved by comparing their absorbance with a reference curve prepared with known standard concentrations included in the kit. Detected antibody was inversely proportional to catecholamine concentrations of the sample.

### First 24-hour events

All significant clinical/surgical events during the first 24 hours were recorded, including any treatment with vasoactive drugs, neurosurgical procedures, hypotension and intracranial hypertension episodes, respiratory failure, changes in chest radiography, electrocardiogram and head CT.

### Outcome assessment

The primary outcome was the association between circulating catecholamine levels measured on hospital admission with mortality and functional outcome assessed by the extended Glasgow Outcome Scale (GOSE) at 6 months. The outcome assessment was performed by structured telephone interviews [11, 12] with the patient or his/her caregiver. The interviewers were blinded to the patients’ catecholamine levels. For the analysis, GOSE outcome was dichotomized into favorable (GOSE 5–8) and unfavorable (GOSE 1–4) outcome (Additional file 1: Table S2).

### Statistical analyses

Demographic and clinical characteristics were summarized to compare patients who experienced unfavorable outcome with those who had favorable outcome using mean ± standard deviation (SD) for continuous variables, or frequency distributions and percentages for categorical variables. Statistical differences were assessed with one-way ANOVA, Mann–Whitney *U* or *X* ^2^ tests as applicable. Bar charts were plotted to examine changes in catecholamine levels over the first 24 hours post-injury. Fractional polynomial plots were obtained to investigate the relation of catecholamine levels to severity of brain injury on CT scans based on the Marshall scoring system. The association between baseline catecholamine levels and outcome at 6-month follow-up was investigated by fitting fixed-effects logistic regression models. An adjusted analysis accounted for the core prognostic factors of TBI including age, GCS score, pupillary reactivity and Marshal CT score of brain injury [10]. Logarithmic transformation was performed to correct for the extreme skewness in the distribution of catecholamine values prior to inclusion in the regression models. Furthermore, we investigated the effect of temporal changes in catecholamine levels on outcome using a random-effects logistic regression model to disaggregate the within-subject effect of catecholamine, which reflects the effect of the temporal changes in catecholamine levels, from the between-subject effect of catecholamine. Effect size was reported as the odds ratio (OR) associated with one logarithmic change in catecholamine level. Statistical significance was set at a *p* value <0.05. All data were analyzed using GraphPad Prism Version 6.0d (GraphPad Inc., San Diego, CA, USA) and Stata version 13.1 (StataCorp, College Station, TX, USA).

## Results

The demographic, clinical, and outcome variables of the participants are shown in Table 1. One hundred and seventy-four patients with isolated blunt TBI were enrolled into the study. The mean age of the patients was 47.5 ± 21.1 years; most were men (75%). The mean time from injury to hospital admission was 80 ± 66 minutes.

**Table 1:** Baseline characteristics of the study cohort

Characteristic	All patients	Outcome at 6 months		*p* value
		Favorable (GOSE 5–8)	Unfavorable (GOSE 1–4)	
Demographics				
Frequency	174	65 (37.4)	109 (62.6)	--
Age (years)	47.5 ± 21.2	37.5 ± 16.5	53.7 ± 21.4	**<0.001**
Male	130 (74.7)	53 (40.8)	77 (59.2)	0.110
Clinical characteristics				
Time to ED (min)	80.2 ± 66.0	87.2 ± 66.9	76.1 ± 65.4	0.218
GCS on admission	5.9 ± 3.1	7.3 ± 3.2	5.1 ± 2.7	**<0.001**
ISS on admission	24.4 ± 11.6	17.9 ± 9.8	28.4 ± 10.8	**<0.001**
AIS head on admission	4.1 ± 1.1	3.5 ± 1.2	4.5 ± 0.8	**<0.001**
Marshall score				
I	27 (15.5)	19 (70.4)	8 (29.6)	--
II	82 (47.1)	35 (42.7)	47 (57.3)	--
III	13 (7.5)	3 (23.1)	10 (76.9)	--
IV	30 (17.2)	2 (6.7)	28 (93.3)	--
V	21 (12.1)	6 (28.6)	15 (71.4)	--
VI	1 (0.6)	0 (0.0)	1 (100.0)	--
SBP (mmHg)	140.4 ± 32.2	140.9 ± 28.4	140.1 ± 34.6	0.861
SaO_2_ <92%	7 (4.0)	3 (42.9)	4 (57.1)	0.759
Mechanical ventilation	144 (82.8)	45 (31.2)	99 (68.7)	**<0.001**
Neurosurgery	51 (29.3)	11 (21.6)	40 (78.4)	**0.006**
Pupils				
Unequal	46 (26.4)	7 (10.7)	39 (35.7)	**0.0005**
Unreactive	72 (41.3)	14 (21.5)	58 (53.2)	**0.0001**

SBP and SaO_2_ refer to levels on admission. All data represented as either the mean ± standard deviation, or frequency (percent), where appropriate. Bold indicates *p* <0.05 by Student’s independent *t* test or *chi* ^2^, between unfavorable and favorable outcome, where appropriate

*Abbreviations: GOSE* extended Glasgow Outcome Scale, *ED* Emergency Department, *GCS* Glasgow Coma Scale, *ISS* Injury Severity Score, *AIS* Abbreviated Injury Scores, *SBP* systolic blood pressure, *SaO*_*2*_ oxygen saturation

### Temporal changes in catecholamine levels over the first 24 h post-injury

**Fig. 2:**
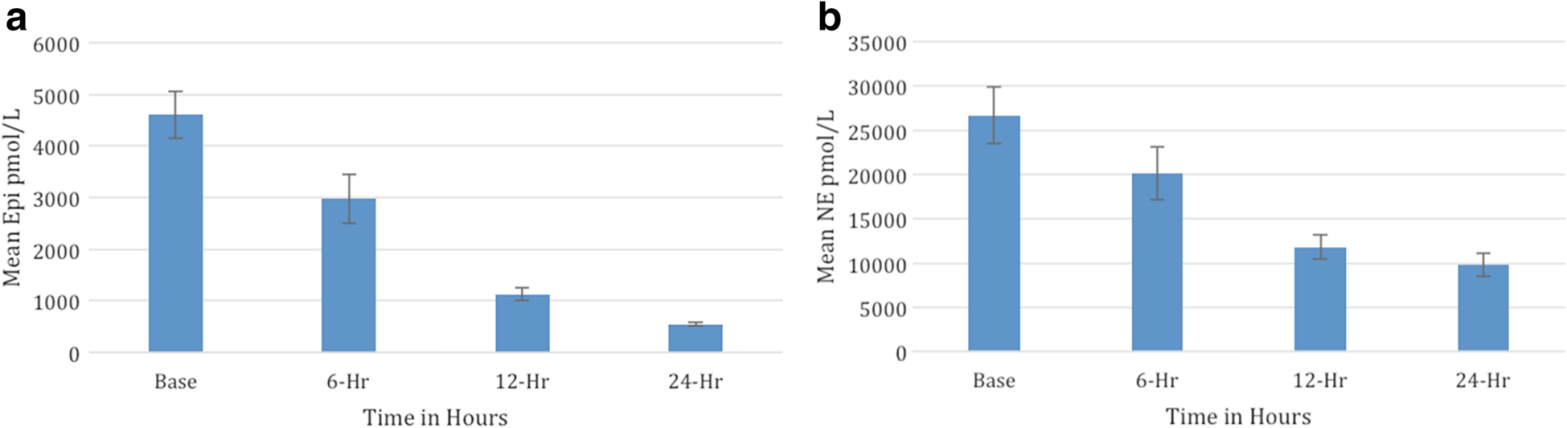
Bar plot showing temporal changes in mean catecholamine levels. **a** Epinephrine levels. **b** Norepinephrine levels

Figure 2 is a bar plot of temporal changes in catecholamine levels over the first 24 h post-injury, including the levels measured in 50 healthy volunteers. The levels of Epi and NE were highest at baseline measurement immediately following admission. The mean levels dropped at a rate of approximately 50% at repeat measurements.

### Association of catecholamine levels and severity of the brain injury

**Fig. 3:**
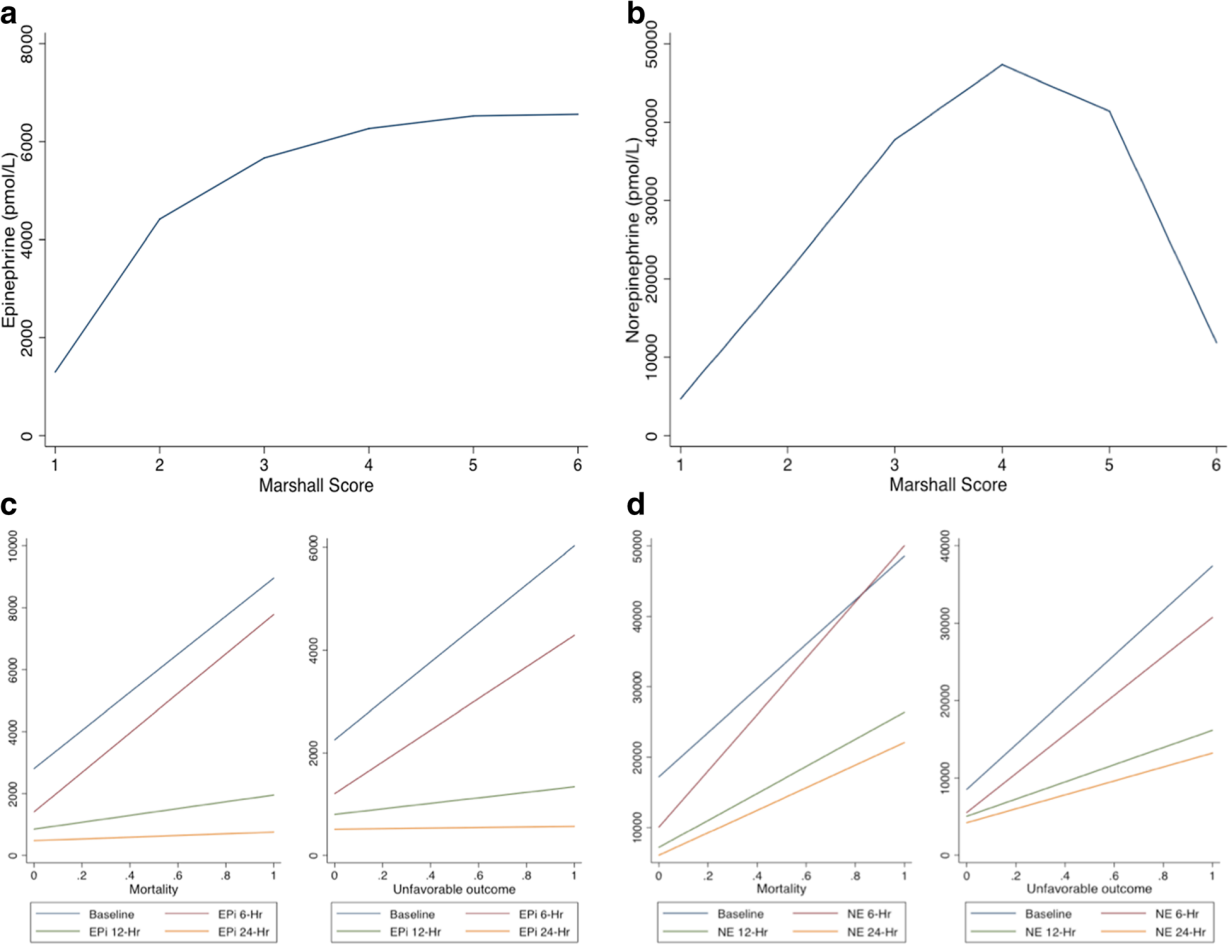
**a** and **b** Fractional polynomial plots of the relation of hospital-admission catecholamine levels to Marshall CT score of brain injury. **c** and **d** Fractional polynomial plots of the relation of catecholamine levels to outcomes at the different time points. *Epi* epinephrine, *NE* norepinephrine

Figure 3a and b shows the relation of baseline catecholamine levels and the Marshall CT score of brain injury severity [10]. Epi levels rose sharply with increasing Marshall score up to a score of 3, and then slowly plateaued beyond a Marshall score of 3 (Fig. 3a). We noted a similar rise in NE levels with higher Marshall scores. However, NE levels dropped sharply beyond a Marshall score of 4 (Fig. 3b).

### Effect of baseline cathecolamine levels on outcome

At 6 months, 109 patients (62.6%) had an unfavorable outcome, including 51 deaths (29.3%). Sixty-five patients (37.4%) had a favorable outcome and two patients (1.1%) were lost in the follow-up. Compared with patients who experienced favorable outcomes, those with unfavorable outcomes had significantly higher median baseline levels of both Epi (1216 vs. 4280 pmol/L, *p* <0.0001) and NE (5298 vs. 28,492 pmol/L, *p* <0.0001) at admission. The results of a fixed-effect logistic regression analysis are shown in Table 2. In the unadjusted analysis, higher baseline levels of Epi and NE were strongly associated with a higher risk of mortality. In the adjusted analysis, the effect of Epi remained statistically significant [OR, 2.86, 95% confidence interval (CI): 1.62–5.01, *p* = 0.001], but the effect of NE was not statistically significant (1.45, 95% CI: 0.98–2.17, *p* = 0.07). Higher Epi and NE levels were statistically associated with higher risk of unfavorable outcome in the unadjusted analyses (Table 2), and after adjustment for age, GCS, and pupillary reactivity (Epi: OR, 2.04, 95% CI: 1.31–3.18, *p* = 0.002; NE: 1.59, 95% CI: 1.07–2.35, *p* = 0.022).

**Table 2:** Results of fixed-effect logistic regression analysis for the effect of hospital-admission catecholamine levels on 6-month outcome of isolated TBI

Catecholamine	Mortality outcome	*p* value	Unfavorable outcome	*p* value
	OR (95% CI)			
Epi unadjusted	2.90 (1.93–4.36)	<0.001	1.93 (1.41–2.64)	<0.001
Epi adjusted^a^	2.86 (1.62–5.01)	<0.001	1.59 (1.07–2.35)	0.022
NE unadjusted	2.15 (1.55–2.98)	<0.001	2.66 (1.79–3.94)	<0.001
NE adjusted^a^	1.5 (0.98–2.17)	0.070	2.04 (1.31–3.18)	0.002

^a^Adjusted to age, Glasgow Coma Scale and pupillary reactivity

*Abbreviations: OR* odds ratio, *CI* confidence interval, *Epi* epinephrine, *NE* norepinephrine

### Effect of temporal changes in catecholamine levels on outcome

Figure 3c and d shows fractional polynomial plots of the relationship between catecholamine levels and outcomes at the different time points. The graph demonstrates a linear relationship between catecholamine levels and outcome, and a complex interaction with time. Admission Epi and NE levels had the strongest prognostic effect. The effects of Epi and NE on outcome weakened with time. Likelihood ratio tests of one-way interaction with time was significant only for mortality outcome (mortality: Epi, *p* = 0.004; NE, *p* <0.001; unfavorable outcome: Epi, *p* = 0.73; NE, *p* = 0.50). Table 3 shows results from the random-effects logistic regression analysis to examine the effect on outcome of the changes with time in catecholamine levels. The estimated conditional between-subject effects of Epi on mortality and unfavorable outcomes were statistically significant in the unadjusted and adjusted analysis (*p* <0.001). The within-subject effects, which reflects the effects of changes in Epi level with time, was statistically significant for mortality but not for unfavorable outcome in the unadjusted analysis (*p* = 0.02). In the adjusted analysis, the within-subject effects were not significant for mortality (*p* = 0.24) or unfavorable outcome (*p* = 0.87). The estimated conditional between-subject effects of NE on mortality as well as unfavorable outcome were significant in unadjusted and adjusted analysis (*p* <0.001). The within-subject effect of NE was significant for mortality in both unadjusted and adjusted analysis, indicating a drop in NE levels with time was associated with a lower risk of mortality. The within-subject effect was not significant for unfavorable outcome in the unadjusted and adjusted analysis (*p* = 0.86 and 0.69 respectively).

**Table 3:** Results of random-effects logistic regression analysis to examine the effect of temporal changes in catecholamine levels on 6-month outcome of isolated TBI

	Mortality	*p* value	Unfavorable	*p* value
	OR (95% CI)		OR (95% CI)	
Epinephrine				
Unadjusted				
Between-subjects	5.91 (3.58–9.75)	<0.001	2.18 (1.59–2.99)	<0.001
Within-subjects	0.84 (0.73–0.84)	0.02	0.96 (0.87–1.07)	0.47
Adjusted^a^				
Between-subjects	5.99 (2.93–12.22)	<0.001	1.57 (1.06–2.33)	<0.001
Within-subjects	0.89 (0.73–1.08)	0.24	0.99 (0.87–1.13)	0.87
Norepinephrine				
Unadjusted				
Between-subjects	5.14 (3.29–8.03)	<0.001	3.55 (2.43–5.22)	<0.001
Within-subjects	0.82 (0.70–0.96)	0.02	0.99 (0.87–1.13)	0.86
Adjusted^a^				
Between-subjects	3.40 (1.93–5.99)	<0.001	2.34 (1.46–3.73)	0.001
Within-subjects	0.78 (0.63–0.97)	0.02	1.03 (0.88–1.21)	0.69

^a^Adjusted to age, Glasgow Coma Scale and pupillary reactivity

*Abbreviations: TBI* traumatic brain injury, *OR* odds ratio, *CI* confidence interval

## Discussion

In this study, we investigated the timeline of catecholamine release during the initial 24 h post-injury in a large prospective cohort of isolated moderate-to-severe TBI patients and its association with mortality and long-term functional outcome. In total, 174 patients with isolated TBI were enrolled (79% severe and 21% moderate), with an excellent long-term follow-up (99%) at 6 months. Our main findings were: (1) TBI patients displayed a pattern of peripheral catecholamine release over the first 24 h of injury characterized by a massive release into the peripheral circulation early in the course of disease, followed by a gradual decline over time. (2) Both Epi and NE levels on admission demonstrated an independent association with functional outcome, measured by GOSE at 6 months post-injury in a dose–response fashion.

Traumatic brain injury leads to an immediate and profound SNS activation with massive release of both central and peripheral catecholamines [6], which is essential for survival. Chesnut et al. [7] showed that early hypotension (i.e., a single measure of systolic blood pressure <90 mm Hg occurring from injury to the resuscitation period) is associated with a doubling mortality, and hypotension occurring in the intensive care unit (i.e., late hypotension) is associated with 11-fold higher risk of death after severe TBI [13]. When early and late hypotension occurs concurrently, the risk of death or vegetative state approximates 80% [13]. Therefore, while SNS activation is an essential adaptive response after brain injury, when excessive or prolonged, that hyperadrenergic state may have a negative impact on outcome.

Early small studies have already recognized the association between high catecholamine levels and severity of the brain injury, duration of mechanical ventilation, myocardial damage, endocrine abnormalities, length of hospital stay, and functional outcome [6, 14–16]. For example, Clifton et al. [15] evaluated the levels of NE and dopamine in the first week after mild-to-severe TBI in 48 patients (18 severe, 17 moderate, and 13 mild). In patients with isolated TBI, NE levels were proportional to the severity of brain injury, measured by GCS on admission. Alert patients (GCS 14) had normal NE values while patients in coma had levels up to seven times above normal [15]. Additionally, blood pressure, heart rate, and temperature increase were proportional to elevations in serum levels of NE. Our results support and expand the concept that circulating catecholamine levels are proportional to the severity of the brain injury [14, 15]. In our cohort, the catecholamine levels were associated with the degree of brain swelling and midline shift according to the Marshall score [10]. The group including patients with brain swelling and compressed or absent cisterns (Marshall III) and patients with brain swelling and midline shift > 5 mm (Marshall IV) displayed the highest levels of NE at all sampled time points. The Marshall score [10] was developed based on observations made during the pilot phase of the National Traumatic Coma Databank study, which showed that intracranial hypertension became increasingly more frequent according to the mesencephalic cisterns status and the degree of midline shift [17]. The mortality rates were directly related to the status of the basal cisterns on the initial CT scan [10]. Likewise, in our cohort the degree of basal cisterns compression and/or the degree of midline shift were related to the highest catecholamine levels on admission and the highest rates of mortality.

Hamill et al. [14] were the first to describe the role of catecholamines as prognostic biomarkers of head injury. In their cohort of only 33 patients (22 severe and 11 moderate TBI), catecholamine levels were also associated to the severity of brain injury. Patients with GCS 3 or 4 on admission, NE and Epi levels were four- to fivefold above normal levels, while patients admitted with GCS >11 had only slightly elevated or normal catecholamine levels. Interestingly, patients with severe and unchanging neurological status in the first week after injury had markedly elevated NE levels on admission, whereas initial NE levels were only mildly elevated in patients who improved to a GCS >11. The authors concluded that the “levels of circulating catecholamines are excellent endogenous and readily quantifiable markers that appear to reflect the extent of brain injury and that may predict the likelihood of recovery.” In another study, Woolf et al. [16] analyzed the catecholamine response to multisystem trauma. They found NE levels were significantly correlated with severity of injury only if the injury included the brain. The same group had described that patients with high levels of NE (>900 pg/mL) remained in poor clinical status (with a low GCS) or died, while patients with NE levels <900 pg/mL improved to a GCS of 11 within 1 week [6].

Regarding the pattern of catecholamine release into the peripheral circulation, contrary to what Hamill et al. [14] described in their cohort “that catecholamine levels remained relatively stable within the first 48 hours of injury”, our measurements demonstrate that circulating catecholamines display a characteristic pattern of release over the first 24 h of injury. This pattern is characterized by a massive release of NE and Epi into the peripheral circulation, with peak levels detected on admission, followed by gradual decline over the subsequent 24 h. However, these levels remained significantly higher by the end of the 24 h period, when compared to measurements done in healthy volunteers. In addition, when TBI patients were divided into favorable and unfavorable outcome groups, both groups displayed a similar pattern over the first 24 h, and both Epi and NE levels remained significantly higher in the unfavorable outcome group (Fig. 3a and b).

Our present study further demonstrates that admission Epi and NE levels are independently associated with 6-month outcome measured by GOSE. Our analysis seems to suggest that both Epi and NE levels are associated with unfavorable outcome, while the levels of Epi are only related to mortality outcome. A dose–response relationship was seen, with higher catecholamine levels on admission being related to outcome. Though the catecholamine levels decreased with time, we found this temporal change had no relation to outcome; rather it was the absolute level of catecholamines at baseline (admission) that was associated with outcome.

Several possible pathophysiological mechanisms may explain the relationship between high catecholamine levels and worse outcomes after TBI:

1. Animal models of cardiac arrest have demonstrated that Epi injection during cardiopulmonary resuscitation (CPR) has detrimental effects through its alpha-1-adrenergic receptor actions on cerebral microvascular blood flow [18, 19]. It reduces cortical microcirculatory blood flow, which increases the severity of cerebral ischemia during CPR [19], and after restoration of spontaneous circulation [18].
2. The systemic inflammatory response is mediated by the increased catecholamine levels [20]. Additionally, the sympathoadrenal activation drives coagulopathy and endotheliopathy [21], through endothelial damage/dysfunction, mostly glycocalyx disruption. This complex interaction between the SNS, endotheliopathy, inflammation and coagulation remains to be completely understood in the acute phase of TBI [20, 21].
3. Increased cardiac and cerebral oxygen demands [22–25].
4. Hypermetabolism, protein catabolism and muscle wasting [26–29]. Hypermetabolism is a common metabolic response of trauma, and follows in concert the increased sympathetic system activity. It is associated with altered lipid and protein metabolism, leading to loss of lean body mass [30].
5. Increased intracapillary hydrostatic pressure leading to vasogenic cerebral edema [31, 32].

Our findings underscore the need for further studies to determine whether a causal relationship exists between catecholamine levels and clinical outcome after TBI, as such a relationship may represent an opportunity for targeted pharmacological therapy against secondary injury after TBI. Presently, no specific pharmacological treatment exists that effectively prevents or limits the progression of secondary brain injury [33]. Given the present findings, adrenergic blockade, may therefore, be a potential therapeutic intervention worthy of further exploration. A recent meta-analysis has demonstrated that exposure to beta-blockers after TBI was associated with a profound reduction of in-hospital mortality by 65% (pooled adjusted odds ratio 0.35; 95% CI 0.27–0.45) [34]. Despite these results, the benefits of the use of beta-blockers in the acute phase of TBI remain unproven and in need of a more robust evaluation in a randomized clinical trial.

## Conclusions

This multicenter, prospective, observational cohort study has demonstrated that circulating catecholamine levels are markedly elevated in moderate and severe isolated blunt TBI patients. This elevation follows a pattern, characterized by massive release into peripheral circulation early after injury, decreasing thereafter over the first 24 hours. Peak levels of catecholamines are markers of brain injury severity and are independently associated with functional outcome measured by the 6-month GOSE, in a dose-dependent fashion. Also, elevated Epi levels on admission were independently associated with an increased risk of death.

## Additional file

Additional file 1: Additional description of Procedures, Data Collection, and Routine Clinical Hematology Analyses. **Table S1** - CT Classification - Traumatic Coma Data Bank. **Table S2** - Functional Outcome according to the extended Glasgow Outcome Scale. (DOCX 18 kb)

## Abbreviations

AIS: Abbreviated Injury Scores
CI: Confidence interval
CPR: Cardiopulmonary resuscitation
CT: Computed tomography
ED: Emergency Department
Epi: Epinephrine
GCS: Glasgow Coma Scale
GOSE: Extended Glasgow Outcome Scale
ISS: Injury Severity Score
NE: Norepinephrine
OR: Odds ratio
SD: Standard deviation
SNS: Sympathetic nervous system
TBI: Traumatic brain injury
