# Supplementary material for: Catecholamines as outcome markers in isolated traumatic brain injury: the COMA-TBI study
Source: Crit Care. 2017 Feb 23;21:37. doi: 10.1186/s13054-017-1620-6 (PMC5322658; doi:10.1186/s13054-017-1620-6)
Supplement: Additional file 1: — Additional description of Procedures, Data Collection, and Routine Clinical Hematology Analyses. Table S1 - CT Classification - Traumatic Coma Data Bank. Table S2 - Functional Outcome according to the extended Glasgow Outcome Scale. (DOCX 18 kb) [file 13054_2017_1620_MOESM1_ESM.docx]

Procedures and Data Collection

Upon hospital admission, clinical and demographic data were obtained from eligible patients, including:

*Demographics –* age and gender

*Clinical information –*mechanism of injury, elapsed time from the trauma to the emergency room, Injury Severity Score (ISS) and AIS

*Neurological status –* level of consciousness categorized by the GCS, pupil size and reactivity, seizures, alcohol level

*Clinical status –* blood pressure, tracheal intubation, spontaneous vs. mechanical ventilation, oxygen saturation, temperature

*Medical history* – past medical history, present medications, including beta-blockers and anticoagulants.

*Routine laboratory exams* *and imaging* were also completed upon admission, including chest radiography and noncontrat computerized tomography (CT) head scans.

*All significant clinical events during the first 24h* were recorded, including, but not limited to: sepsis, organ failure, any medical treatments administered, surgical procedures, and any other significant changes in clinical parameters. For patients who died, cause of death was recorded and classified as TBI related or non-TBI related. Upon hospital discharge, at 28 days, and at 6-months, patient outcome was assessed using the extended Glasgow Outcome Scale (GOSE) score.

**Routine Clinical Hematology Analyses**

At admission, routine hematological parameters were measured for each patient. These included: complete blood counts, arterial blood gas and lactate, troponin, sodium (Na), potassium (K), glucose, ethanol (ETOH) level, international normalized ratio (INR), and activated partial thromboplastin time (aPTT).

**Table S1 -** CT Classification - Traumatic Coma Data Bank

| **Marshall Classification** | **Computed Tomography Findings** |
| --- | --- |
| **Diffuse injury I (no visible pathology)** | No visible intracranial pathology seen |
| **Diffuse injury II** | Cisterns are present with midline shift of 0-5 mm and/or lesions densities present; no high or mixed density lesion >25 cm^3^, may include bone fragments and foreign bodies. |
| **Diffuse injury III (swelling)** | Cisterns compressed or absent with midline shift of 0-5 mm; no high or mixed density lesion >25 cm^3^ |
| **Diffuse injury IV (shift)** | Midline shift >5 mm; no high or mixed density lesion >25 cm^3^ |
| **Evacuated mass lesion - V** | Any lesion surgically evacuated |
| **Non-evacuated mass lesion - VI** | High or mixed density lesion >25 cm^3^; not surgically evacuated |

**Table S2** – Functional Outcome according to the extended Glasgow Outcome Scale

|  |  | **Functional Outcome** | **Abbreviation** | **Description** |
| --- | --- | --- | --- | --- |
| **Unfavorable Outcome** | 1 | Death | D | Death |
|  | 2 | Vegetative State | VS | Condition of unawarenes with only reflex responses but periods of spontaneous eye opening. |
|  | 3 | Lower severe disability | SD - | Patient is dependent for daily support for daily support, Because of mental or physical disability, or a combination of both. **Patient cannot be left alone for more than 8 h.** |
|  | 4 | Upper severe disability | SD + | Patient is dependent for daily support for daily support, Because of mental or physical disability, or a combination of both. **Patient can be left alone for more than 8 h.** |
| **Favorable Outcome** | 5 | Lower moderate disability | MD - | Patients have some diasability such as aphasia, hemiparesis or epilepsy and/or deficits of memory or personality, however are able to look after themselves.They are independent at home, but dependent outside. **Patient is not able to return to work.** |
|  | 6 | Upper moderate disability | MD + | Patients have some diasability such as aphasia, hemiparesis or epilepsy and/or deficits of memory or personality, however are able to look after themselves. They are independent at home, but dependent outside. **Patient is able to return to work.** |
|  | 7 | Lower good recovery | GR - | Resumption of normal life with the capacity to work even if the pre-injury status has not been achieved. **Patient has minor disabling neurological or psychological deficits.** |
|  | 8 | Upper good recover | GR + | Resumption of normal life with the capacity to work even if the pre-injury status has not been achieved. **Patient has no disabling neurological or psychological deficits.** |

**Reference**

1. Maas AIR, Hukkelhoven CWPM, Marshall LF, Steyerberg EW. Prediction of outcome in traumatic brain injury with computed tomographic characteristics: a comparison between the computed tomographic classification and combinations of computed tomographic predictors. Neurosurgery. 2005 Dec.;57(6):1173-82.
2. Marshall LF, Gantille T, Klauber MR, et al.: The outcome of severe closed head injury. J Neurosurg. 1991 (Suppl)75:28-36.
